# Proteogenomic analysis of pitaya reveals cold stress-related molecular signature

**DOI:** 10.7717/peerj.8540

**Published:** 2020-02-11

**Authors:** Junliang Zhou, Zhuang Wang, Yongya Mao, Lijuan Wang, Tujian Xiao, Yang Hu, Yang Zhang, Yuhua Ma

**Affiliations:** 1 Guizhou Institute of Pomological Sciences, Guizhou Academy of Agricultural Sciences, Guiyang, Guizhou, China; 2 Zhejiang Academy of Forestry, Hangzhou, Zhejiang, China; 3 Zhejiang Provincial Key Laboratory of Biological and Chemical Utilization of Forest Resources, Hangzhou, Zhejiang, China; 4 Fudan University, Institutes of Biomedical Sciences, Shanghai, Shanghai, China

**Keywords:** Transcriptome, Shotgun label-free Proteomic, Pitaya, Cold stress, Proteins expression

## Abstract

Pitayas (*Hylocereus* spp.) is an attractive, highly nutritious and commercially valuable tropical fruit. However, low-temperature damage limits crop production. Genome of pitaya has not been sequenced yet. In this study, we sequenced the transcriptome of pitaya as the reference and further investigated the proteome under low temperature. By RNAseq technique, approximately 25.3 million reads were obtained, and further trimmed and assembled into 81,252 unigene sequences. The unigenes were searched against UniProt, NR and COGs at NCBI, Pfam, InterPro and Kyoto Encyclopedia of Genes and Genomes (KEGG) database, and 57,905 unigenes were retrieved annotations. Among them, 44,337 coding sequences were predicted by Trandecoder (v2.0.1), which served as the reference database for label-free proteomic analysis study of pitaya. Here, we identified 116 Differentially Abundant Proteins (DAPs) associated with the cold stress in pitaya, of which 18 proteins were up-regulated and 98 proteins were down-regulated. KEGG analysis and other results showed that these DAPs mainly related to chloroplasts and mitochondria metabolism. In summary, chloroplasts and mitochondria metabolism-related proteins may play an important role in response to cold stress in pitayas.

## Introduction

Pitaya (*Hylocereus* spp.), a member of the Cactaceae family, has been attracting attention worldwide owing to their attractive, highly nutritious and commercially valuable fruits (Luo et al., 2014; Hua et al., 2016), which can be found in the “small exotic fruits” category in the fruit market. Two types of pitayas are commercially produced on a large-scale: the white-fleshed pitaya (*Hylocereus undatus*) and the red-fleshed pitaya (*Hylocereus polyrhizus*) (Song et al., 2016a, 2016b). The *Hylocereus* genus used to grow at the subtropical and tropical regions of the Americas and now pitaya crops are mainly grown in countries such as Colombia, Mexico, Costa Rica, Nicaragua and Vietnam (Ortiz & Takahashi, 2015). The pitaya plant has also attracted horticultural interest because it is highly drought resistant, enabling pitayas to be grown in areas stricken by drought (Rodriguez-Jimenez et al., 2014; Jimenez et al., 2012). In South China, the pitaya plantations are thriving, particularly in the karst regions, such as the Yunan and Guizhou provinces.

In the past decades, pitaya research studies have mainly focussed on the biochemistry of the betalains synthesized by pitaya, including their purification and identification (Stintzing, Schieber & Carle, 2002; Wybraniec et al., 2009; Sogi et al., 2010), their physical and chemical properties (Woo et al., 2011), and their antioxidant and radical-scavenging capacity (Garcia-Cruz et al., 2013). Metabolite profiling of red-fleshed (*H. polyrhizus*) and white-fleshed (*H. undatus*) pitayas has tentatively identified several betalain biosynthesis-related compounds (Suh et al., 2014). Several key genes in the betalain biosynthesis pathway have also been identified using transcriptomic analysis (Hua et al., 2016). The *HuCAT3* gene of pitaya, which encodes catalase, has been isolated and characterized and its expression profile under abiotic stress has been analyzed (Nie et al., 2015). However, the genomic resources available for pitaya are still scarce. More genetic data needs to be generated to aid further studies, such as investigations of pitaya resistance to abiotic and biotic stresses, and for crop breeding.

High-throughput-omics techniques like genomics, transcriptomics or proteomics have recently been widely adopted by plant biologists for studying the plants under varies of different environmental stress (Meena et al., 2017). Especially, high-throughput RNA sequencing (RNA-Seq) technology is a powerful and cost-efficient tool for transcriptome analysis (Ansorge, 2009; Sa et al., 2013; Yu et al., 2014; Wang et al., 2010). For gene expression profiling, especially in those organisms that are non-model organisms and lack genomic sequences, RNA-Seq is a particularly suitable technology. For example, Illumina sequencing technology offers millions of sequence reads from a single instrument run, and only takes a few days to generate a huge amount data (Crawford et al., 2010). It has been shown that the sequencing data from a single illumina run can generate enough read coverage for de novo transcriptome assembly and gene discovery and differential expression profiling analysis (Hudson, 2008). However, mRNA is biological intermediate product, which cannot substantially reflect protein expression level. Recently, tandem mass spectrometry coupled with high performance liquid chromatography have provided a way for obtaining global proteome data and their expression, named label-free proteomics methods. Label-free proteomics have been successfully applied in many plants such as *Arabidopsis thaliana* (Niehl et al., 2013) and *Nicotiana attenuata* (Weinhold et al., 2015), and non-model plants like *Zingiber zerumbet* (Mahadevan et al., 2015) and *Piper nigrum* (Mahadevan et al., 2016). Label-free proteomics is a high-throughput technique, which has several advantages including handling of proteins without gels, in-solution digestion by trypsin and easy use of internal peptide standards. Label-free proteomics is also applicable to identification of novel proteins and studying non-model organism proteomes which have very limited genomic information (Mahadevan et al., 2016).

In this study, we used Illumina sequencing technology to sequence the transcriptome of pitaya. The unigenes were annotated using six public databases (UniProt, Pfam, InterPro, KEGG and NR and COGs at NCBI), and served as the background database for label-free proteomics of pitaya. Here, we focused on differentially abundant proteins (DAPs) between control samples and cold treatments.

## Materials and Methods

### Plant material and RNA extraction

The pitaya cultivar “Tiegusu” was used in this study. “Tiegusu” has light-green flowers and is one of most widely grown commercial cultivars in China. The pitaya plants were grown in a greenhouse at the Guizhou Academy of Agricultural Sciences (Guizhou, China) under a temperature range of 23–28 °C and natural light. To identify as many transcripts involved in cold stress as possible, RNA was extracted from 12 samples of six different tissues (young roots, tender shoots, stems, flower buds, new stems and fruits) from plants that had been grown at 0 °C (cold treatment) for 3 days or grown at a normal temperature (25 °C treatment). A TRIzol^®^ reagent (Invitrogen, Carlsbad, CA, USA) was used to isolate total RNA from each sample and RNase-free DNase I (TaKaRa) was used to treat the samples by following the manufacturer’s protocol. Equal amounts of total RNA from each treatment were pooled together for cDNA library construction and Illumina deep sequencing.

### cDNA library preparation and Illumina sequencing for transcriptome analysis

RNA-Seq was performed at Shenting Genomics Institute (Hangzhou, China) using an Illumina HiSeq™ 2000 (Illumina Inc., San Diego, CA, USA). Briefly, poly (A)^+^ mRNA was isolated from the pooled total RNA sample using Oligo (dT) magnetic beads. The mRNA was fragmented into short fragments using a fragmentation buffer to avoid priming bias. A SuperScript double-stranded cDNA synthesis kit (Invitrogen, Carlsbad, CA, USA) with a random hexamer-primer (Illumina) was used to synthesize double-stranded cDNA. The synthesized cDNA was then subjected to end-repair and phosphorylation, and the repaired cDNA fragments were 3′-adenylated with Klenow exo- (3′–5′ exo minus, Illumina). The ends of the 3′-adenylated cDNA fragments were ligated by Illumina paired-end adapters. The ligation products were purified on 2% agarose gel to select the appropriate templates for downstream enrichment. The cDNA fragments (approximately 200 bp) were excised from the gel. After end reparation and ligation of the adaptors, the products were amplified by PCR using PCR primers PE 1.0 and 2.0 (Illumina) with fusion DNA polymerase and purified using the QIAquick PCR Purification Kit (Qiagen, Valencia, CA, USA). Finally, the cDNA library was constructed using 200 bp insertion fragments. The library was validated using an Agilent Technologies 2100 Bioanalyzer (Agilent Technologies, Palo Alto, CA, USA), then sequenced using Illumina HiSeq™ 2000 (Illumina Inc., San Diego, CA, USA). The raw data was deposit at www.iprox.org with accession project ID: IPX0001296002.

### Data filtering, de novo assembly and annotation

The raw reads were cleaned by removing adaptor sequences, empty reads and low-quality reads using Trimmomatic (v0.32). The cleaned reads (a minimal sequence length of 200 bp with identity value above 95%) were assembled by the de novo software Trinity (v2.0.6) with default parameter. Then the TGICL and Phrap Clustering tools were used to obtain sequences that could not be extended at either end. The obtained sequences were defined as unique transcripts (or unigenes) (Grabherr et al., 2011; Pertea et al., 2003; Vogel et al., 2006). To obtain annotation of transcripts, all unigene sequences were searched against NCBI nr (non-redundant protein) database using the BLASTX algorithm (http://www.ncbi.nlm.nih.gov/) with cutoff of *E*-value 10^−5^. Functional annotation was performed using Gene Ontology (GO) and analyzed using the Blast2go (http://www.blast2go.org) software (Conesa et al., 2005). The COG and KEGG Orthology (KO) annotations were performed using the *A. thaliana* and *Oryza sativa* genome sequences as reference data in the Kyoto Encyclopedia of Genes and Genomes (KEGG) (Kanehisa et al., 2012; Wixon & Kell, 2000; Ogata et al., 1999). HMMER (http://hmmer.org) was used to obtain a domain-based annotation with Pfam (http://Pfam.sanger.ac.uk) database (Finn et al., 2016). All unigene sequences were further scanned by TransDecoder (v2.0.1) to identify Coding sequence (CDS). In totally, 44,337 CDS were predicted.

### Protein extraction and profiling

Each replicate of pitaya samples was pulverized with mortar and pestle to a fine powder in liquid nitrogen, respectively. About 1 g of sample was used for protein extraction using a filter-assisted sample preparation method. Briefly, the sample was suspended in five mL protein extraction buffer (0.5 M Tris–HCl (pH 7.5), 0.7 M sucrose, 0.1 M KCl, 50 mM EDTA, and 40 mM dithiothreitol (DTT)) for 10 min at room temperature. After that, equal volume (five mL) of Tris-phenol was added. After 30 min of shaking, the suspension was centrifuged at 8,000×g and 4 °C for 5 min. After centrifuging, the upper phenolic phase was collected for further extraction by adding an equal volume of extraction buffer to the supernatant. Then, four volumes of 0.1 M ammonium acetate in methanol were added and kept the mixture overnight at −20 °C for protein precipitation, then centrifuging at 4 °C, 8,000×g for 10 min and discarded the supernatant. The pellet was washed thrice at 4 °C with ice-cold acetone. Finally, the pellet was dried for 2 h in a vacuum drier. After drying, the rehydration solution (100 μL; 8 M (w/v) urea, 0.1 M (w/v) Tris, and 10 mM DTT) was used to solubilize the pellet. The concentration of protein was determined by Bradford method.

In the centrifuge tube, the deposit was buffer exchanged with 8 M urea containing 10 mM DTT and 100 mM Tris–HCl. Then, the deposit was alkylated with 55 mM iodoacetamide. The urea concentration in the extract was then diluted to 1 M using the Tris–HCl buffer (pH 7.6). Protein samples were digested by trypsin (enzyme to substrate ratio = 1:20) in a thermomixer (1,000 rpm) overnight at 37 °C. Nano LC-1DTM plus system (Eksigent, Dublin, CA, USA) combined with AB Triple TOF 5600 MS (Foster City, CA, USA) were used to analysis digested peptides. Firstly, eight μL crude polypeptide was injected using a full sample loopin. Crude polypeptide was then desalted on a ChromXP trap column (NanoLC TRAP Column, 3 μm C18-CL, 120 A, 350 μm × 0.5 mm; CA, USA) and then eluted into a second analytical column (Nano LC C18 reversed-phase column (3C18-CL, 75 μm × 15 cm; CA, USA)) using a linear gradient formed by mobile phases A (5% ACN and 0.1% FA) and mobile phases B (95% ACN and 0.1% FA) for 120 min gradient at a flow rate of 300 nL/min. AB Triple TOF 5,600 system was operated in data-dependent acquisition mode to automatically switch between TOF–MS and product ion acquisition using Analyst Software (TF1.6). β-Galactosidase digestion was used to calibrate every two samples by 10 min for elution and 30 min for identification.

### Proteome data processing

Raw MS files from AB Triple TOF 5600 were processed by MaxQuant version 1.5.2 (http://www.maxquant.org). MS/MS spectra were searched against the transcriptome derived database above mentioned. Precursor mass and fragment mass were identified with an initial mass tolerance of 6 ppm and 20 ppm, respectively. The search included variable modifications of methionine oxidation and N-terminal acetylation, and fixed modification of carbamidomethyl cysteine. Minimal peptide length was set to seven amino acids and a maximum of two mis-cleavages was allowed. MS runs were analyzed with the “match between runs” option. For matching, a retention time window of 20 s was selected. Proteins matching to the reverse database were filtered out. The false discovery rate was set to 0.01 for peptide and protein identifications. The raw data of proteome were all deposit at http://www.iprox.org with accession project ID: IPX0001296001.

## Results

### Illumina paired-end sequencing and de novo assembly of reference pitaya transcripts

**Figure 1:**
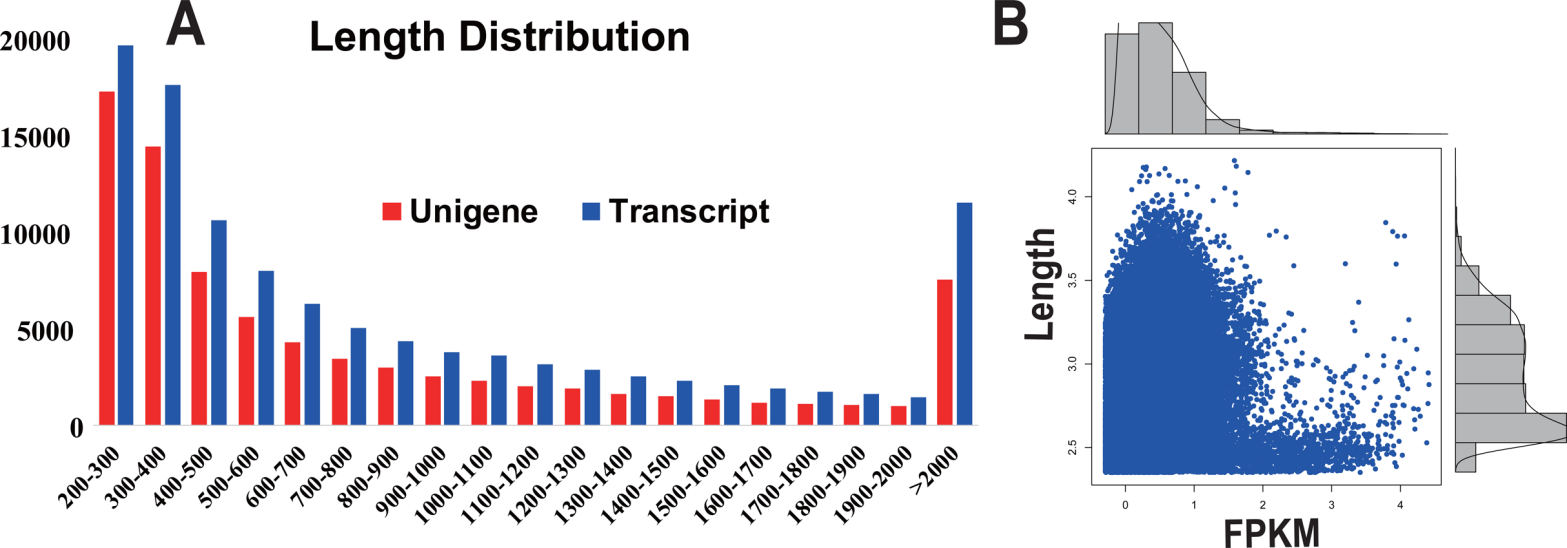
Transcript and unigene length distribution; and the distribution of the FPKM value corresponding to the distribution of unigene length. (A) Transcript and unigene length distribution. The *x*-axis represents the sequence length in base pairs; the *y*-axis represents the number of transcript (blue) and unigenes (red) relative to the sequence length, respectively. (B) The distribution of the FPKM value corresponding to the distribution of unigene length. The *x*-axis represents the sequence length in base pairs; the *y*-axis represents the FPKM value.

In this study, from one plate (8 lanes) in a single sequencing run, a total of 25.3 million reads were obtained, generating approximately 4.2 giga base pairs (Gbp) of raw data (Table 1). After the removal of adaptor sequences, low-quality reads (Q-value < 25) and ambiguous reads, 21.3 million high-quality clean reads (3.1 Gbp, 84.4% of the raw data) remained. The quality of the clean reads data was assessed based on the base-calling quality scores. The scores obtained using Illumina’s base-caller Bustard. Phred-like quality scores at the Q30 level were obtained for 97.23% of the clean reads data. All the high-quality reads were assembled into 110,330 isotigs (81,252 unigenes) with a maximum size of 200 bp (Fig. 1A). The isotigs were more abundant than those previously reported for a pitaya transcriptome dataset by Hua et al. (2016). The greater number of isotigs obtained in our study may be the result of trying to acquire the most comprehensive coverage possible by sequencing RNA extracted from six different types of pitaya plant tissues. The assembled isotigs have an average contig length of 934 bp (868 bp, unigene) and an N50 of 1,445 bp (1,373, unigene) (i.e., 50% of the assembled bases were incorporated into contigs of 1,445 bp or longer). Although a large proportion of the contigs (48.77%) were between 200 to 500 bp, we obtained 62,413 contigs (51.23%) that were more than 500 bp in length (Fig. 1A). Most of the highly expressed unigenes were approximately 2,500 bp in length (Fig. 1B).

**Table 1:** Overview of the sequencing and assembly.

Type	Number
Total raw reads	253,022,990
Total clean reads	213,482,666
Q30 percentage	97.23%
N percentage	0.00%
Total number of contigs	110,330
Total number of contigs (≥500 bp)	62,510
Total number of contigs (≥1,000 bp)	34,973
Total length of contigs (bp)	103,058,657
Mean length of contigs (bp)	934
Largest isotig (bp)	16,440
Smallest isotig (bp)	224
N50 of contigs	1,445
Total number of unigenes	81,252
Total number of contigs (≥500 bp)	41,693
Total number of contigs (≥1,000 bp)	22,696
Total length of unigenes (bp)	70,550,078
Mean length of unigene (bp)	868
Largest unigene (bp)	16,440
Smallest unigene (bp)	224
N50 of unigene	1,373

### Functional annotation and classification of the pitaya transcripts

**Figure 2:**
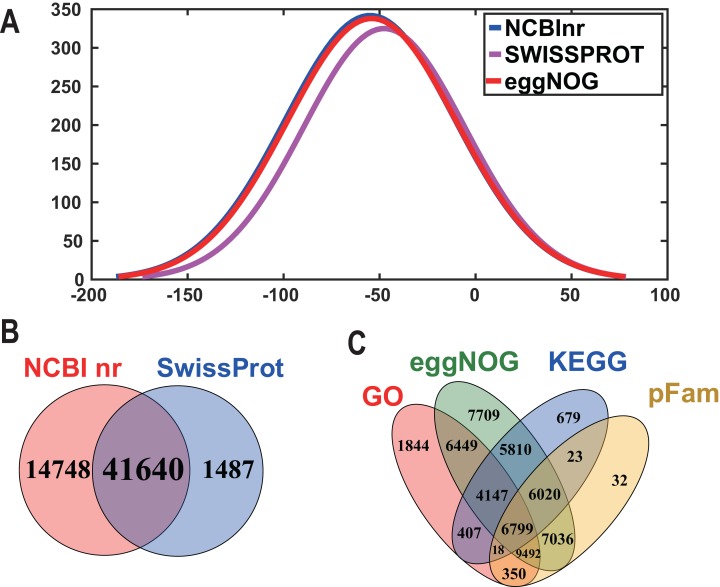
The frequency distribution of the log10 (e-value) of the Nr blast, Swiss–Prot and eggNOG searches and venn diagram of the sequences annotated by different methods. (A) The frequency distribution of the log10 (e-value) of the Nr blast, Swiss–Prot and eggNOG searches. The *x*-axis represents the log10 (*e*-value) of the Nr blast, Swiss–Prot and eggNOG searches; the *Y*-axis represents the frequency. (B) and (C) Venn diagram of the sequences annotated by different methods. (B) Venn diagram of sequences annotated using the NCBI nr and Swiss-Prot databases. (C) Venn diagram of sequences annotated using the GO, eggNOG, KEGG and pFam databases.

**Figure 3:**
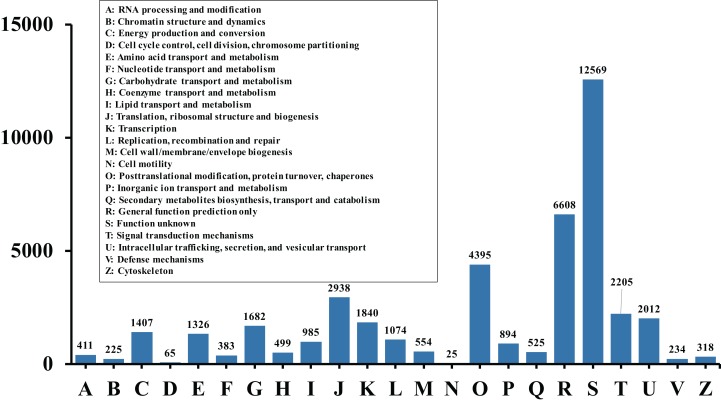
Histogram of KOG classification. All the contigs were aligned with genes in the KOG database to predict and classify possible functions. Of the 56,388 contigs with nr hits, 53,462 sequences were grouped into 23 KOG classifications.

**Figure 4:**
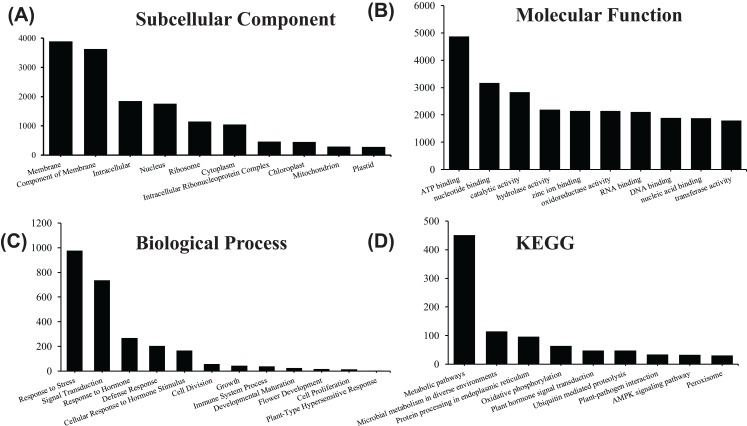
GO and KEGG pathway enrichment analysis of the pitaya transcriptome. Gene number of subcellular location (A) , molecular function (B) and biological process (C) for all identified RNA in pitaya. KEGG pathway mapping result was shown in (D).

**Figure 5:**
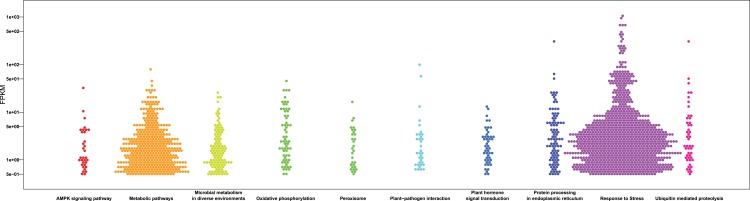
Bee swarm graphs of the enriched KEGG pathway and the FPKM value of the genes within the pathway. The *x*-axis represents the different KEGG pathways, the *y*-axis represents the FPKM value and the hexagons represent genes.

In order to acquire complete functional information, sequence-based alignments of the unigenes were performed against different public databases, including the NCBI non-redundant protein database (NCBInr), Swiss-Prot/UniProt, KEGG pathway, GO and KOG cluster using the BLASTX algorithm with a significant *E*-value threshold of < 10^−5^. The distribution of the log10 (*e*-value) of the NCBInr BLAST and Swiss-Prot searches were normally, and the center of distribution were all at approximately 10^−50^ (Fig. 2A), which indicated that the annotation had a high degree of similarity with known sequences. The Hidden Markov Model method was used to search both the InterPro and Pfam databases for Domain/family searches, and BLASTX was used to search alignments against the Clusters of Orthologous Groups (COGs) database at NCBI, with the *E*-value thresholds also set at ≤ 1 e^−5^ (Table S1). Out of 56,388 hits in the Nr database, 43,127 unigenes also had hits in the Swiss-Prot database (1,487 unigenes only had hits in Swiss-Prot) (Fig. 2B). The number of sequences that were annotated by searching the GO, KEGG and Pfam databases are shown in Fig. 2C. In total, 53,462 sequences were assigned to 23 of the EuKaryotic Orthologous Group (KOG) categories (Fig. 3). The KOG tool is a eukaryote-specific version of the COG tool for identifying orthologous and paralogous proteins (26). The KOG database also provides information about the classification of gene products, including their evolutionary relationships (Tatusov et al., 2003; Koonin et al., 2004). Based on the assumption that every protein evolved from an ancestor protein, pitaya isotigs were compared with known sequences in the KOG database to predict and classify their possible functions. Among the 23 KOG categories, “S: Function unknown” was the largest group (12,569 isotigs; 23.51% of all isotigs), followed by “R: General function prediction only” (6,608; 12.36%) and “O: Posttranslational modification, protein turnover, chaperones” (4,395; 8.2%). “N: Cell motility” was the smallest group (25; 0.04%), followed by “D: Cell cycle control, cell division, chromosome partitioning (65; 0.12%), “B: Chromatin structure and dynamics” (225; 0.42%) and “V: Defence mechanisms” (234; 0.43%) (Fig. 3). To retrieve function of pitaya, we mapped the annotated sequences to GO and canonical KEGG pathways (Kanehisa et al., 2008). The results showed that the most highly represented GO term was the “GO:0006950, response to stress,” with 451 members, and “path:ko04626, plant–pathogen interaction” was enriched at KEGG pathways, with 114 members (Figs. 4D and 5).

### Proteomics characterization of pitaya under cold stress

**Figure 6:**
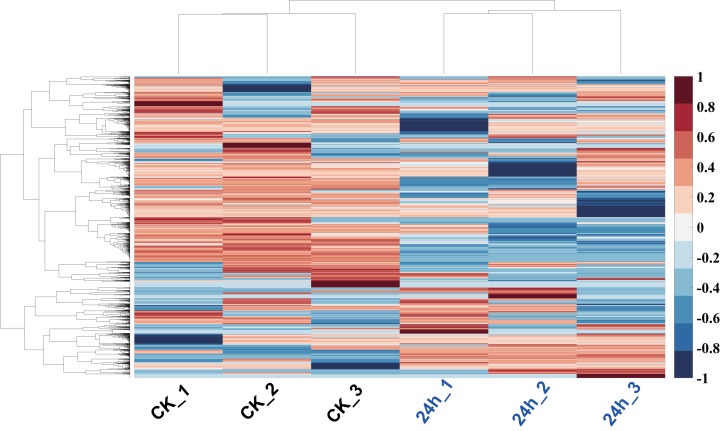
Hierarchy clustering of all profiled proteins. Clustering was based on euclidean distances with average linkage. Each row of protein quantitation value was scaled into the region (−1, 1). Samples of CK were in blue color, and 24 h cold treated samples were in red color.

In order to analyze the mechanism of pitaya response to cold stress, proteomics approach was used to identify and determine the abundance of proteins in all of tissues. The proteins of those samples (cold treatment vs. control) were extracted and each group treatment conducted three biological replicates. iBAQ values derived from MaxQuant software were used to represent the abundance of identified protein. Hierarchy clustering analysis, a quality-control measure based on expression profiles among replicates, indicates that the results are highly reproducible (Fig. 6). A total of 1,712 non-redundant proteins were identified (including those samples with 24 h cold treatment and control; Table S2).

### Comparison of the abundance profiles of proteins between control and cold treatment of pitaya

**Figure 7:**
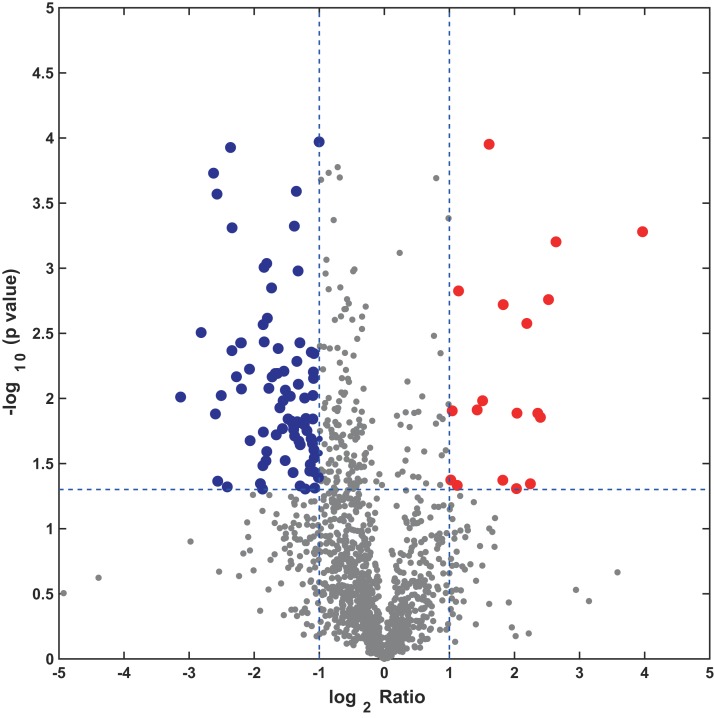
Volcano plot of all profiled proteins. The *x*-axis is log2 based fold change and *y*-axis represent the negative log10 of the *p*-value calculated from two tailed *t*-test. The red points are significant up-regulated proteins, while the blue points are significant down-regulated proteins.

**Figure 8:**
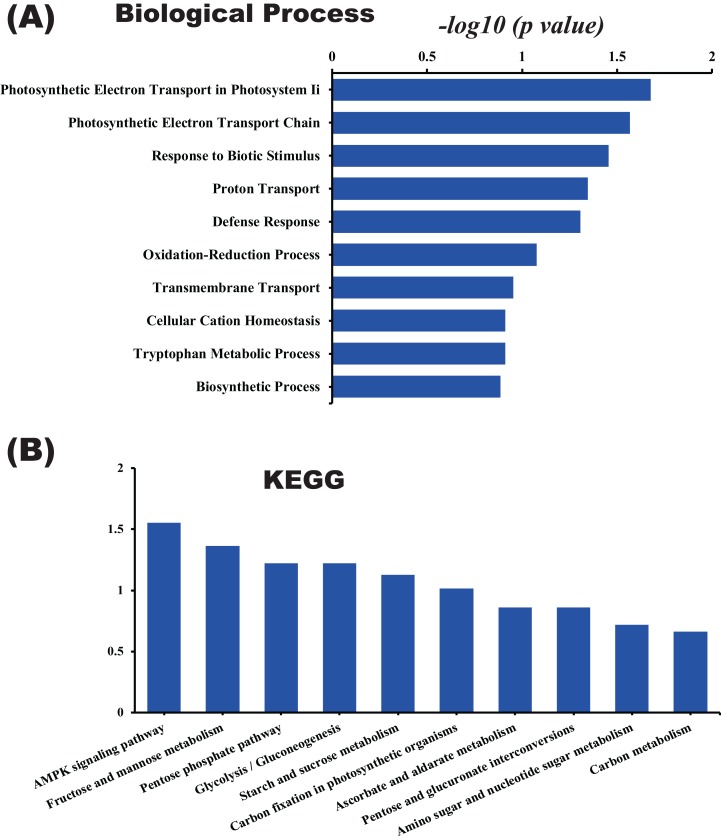
GO and KEGG pathway enrichment analysis of the pitaya proteome. Enrichment score of biological process (A) and KEGG pathway (B) for all identified proteins.

In this study, the label-free quantitative proteomic analysis characterized the differences in protein synthesis between cold treatment and control. We used volcano plot to show differential expressed proteins. Compared with the control group, the up-regulated and down-regulated proteins (fold change ≥ 2, *p* ≤ 0.05) in cold treatment group were 18 and 98, respectively (Fig. 7; Table S3). Total 116 DAPs were subjected into Blast2GO and sorted by major enrichment of biological processes (Fig. 8), including proton transport, ATP hydrolysis coupled proton transport, glycolytic process, carbon fixation, ATP synthesis coupled proton transport, ATP metabolic process, proteolysis, metabolic process, ion transport, and intracellular protein transport. All DAPs were successfully annotated with KEGG pathways, sorted by enrichment score (Fig. 8), including AMPK signaling pathway (path:ko04152), fructose and mannose metabolism (path:ko00051), pentose phosphate pathway (path:ko00030), glycolysis/gluconeogenesis (path:ko00010), starch and sucrose metabolism (path:ko00500), carbon fixation in photosynthetic organisms (path:ko00710), ascorbate and aldarate metabolism (path:ko00053), pentose and glucuronate interconversions (path:ko00040), amino sugar and nucleotide sugar metabolism (path:ko00520), carbon metabolism (path:ko01200). It was found that proteins under ATP related GO catalogues were decreased, which indicated that the cold stress reduced the activity of energy generate, storage or release, which was one of the key aspects of life activity.

### Overview and analysis of differentially abundant proteins related to cold stress

Compared with the control group, there are 18 proteins were increased expression abundance after 24 h cold treatment. The expression level of VPS15 Serine threonine-kinase, TRPA2 alpha chloroplastic tryptophan synthase and PPDK1 chloroplastic phosphate dikinase were significantly higher than other proteins. However, majority of the DAPs were down regulated and the 20 most decreased DAPs were listed in the Table S3. Among them, it is interesting that a number of them are chloroplastic or mitochondrial related proteins. Of which, 6 DAPs, including Ketol-acid reductoisomerase, Glutamate decarboxylase, Malate dehydrogenase, Pentatricopeptide repeat-containing protein, Phosphoglycolate phosphatase and Monodehydroascorbate reductase, are related to chloroplastic proteins, and 5 DAPs, including Aldehyde dehydrogenase family 2, Monodehydroascorbate reductase, Glycine dehydrogenase, and Probable mitochondrial-processing peptidase are related to mitochondrial proteins. Some studies provide evidence that chloroplastic and mitochondrial proteins play an important role in response to cold stress.

## Discussion

The RNA-Seq technology is an efficient technology for characterizing transcriptomes of the non-model organisms. In this study, the isotigs were more abundant than those previously reported for a pitaya transcriptome dataset by Hua et al. (2016), indicating that the Illumina paired-end sequencing project generated a substantial fraction of pitaya genes, so it can be used for next analysis. Interestingly, more and more transcriptome studies have found that the number of up-regulated proteins response to cold stresses was significantly more than down-regulated proteins in wheat (Winfield et al., 2010), *Lotus japonicas* (Calzadilla et al., 2016), and *Arabidopsis* (Fowler & Thomashow, 2002). Many tropical plants adopted in temperate climate including pitaya cannot live in the cold climate. Here, we identified several DAPS refer to cold stress in pitaya and further analysis showed that chloroplasts and mitochondria metabolism-related proteins may play a vital role in response to cold stress in pitayas.

Serine threonine-kinase is one of the protein kinases composed of two-component signal transduction systems (Zschiedrich, Keidel & Szurmant, 2016). The two-component systems are the signal transduction system which most have been investigated at present and play the major roles in regulating cell activities in many eukaryotes. Due to increasing experimental data, it has been discovered that more and more co-regulation and crosstalk regulations among signal transduction systems. And the two-component systems are found that to be very important in corresponding to abiotic stresses (Liu et al., 2015; Tang et al., 2017). The role of serine/threonine protein kinases (STPKs) in the cold response was much studied in *Synechocystis* (Zorina et al., 2014). A screening of a collection of STPK mutants identified it as a possible transcriptional regulator for lower temperatures adaption (Zorina et al., 2014). TRPA2 tryptophan synthase and PPDK1 phosphate dikinase are chloroplastic enzymes. The tryptophan (Trp) biosynthetic pathway leads to the production of many secondary metabolites with diverse functions, *Arabidopsis* tryptophan pathway enzymes have been shown inducing by abiotic stress to allow for increased biosynthesis of secondary metabolites (Zhao, Williams & Last, 1998), which may be the reason of TRPA2 tryptophan synthase increased under cold stress as well. PPDK reversibly interconverts pyruvate, ATP, and orthophosphate with phosphoenolpyruvate (PEP), AMP, and pyrophosphate (PPi) and provides diverse functions in various plant tissues (Lappe et al., 2018). There is a study suggested that PPDK1 was associated with the antioxidant systems (Xu et al., 2018). However, the role of TRPA2 and PPDK1 in cold stress remain unclear yet.

Many metabolic reactions of plant were take place in the chloroplast in plants, however the metabolic balance in chloroplasts is easily disturbed by environmental stresses. Thereby, the reprogram of specific cold-stress proteins in the chloroplast is important for plants adaptation to cold stress (Artus et al., 1996), such as increasing the stability of chloroplast membranes during freezing (Steponkus et al., 1998), modification of photosystem II photochemical properties (Hurry & Huner, 1992) and of ROS-scavenging systems (Bowler & Van Montagu, 1992), resulting in the reduction of sensitivity to photoinhibition at low temperature. In addition, mitochondrial also can produce ROS and many metabolites, which may serve as retrograde signals to adapt cold responses (Ng et al., 2014). A defect in the mitochondrial complex I also enhances ROS accumulation and causes the mutant plants to have reduced expression of cold-responsive genes and to exhibit chilling and freezing sensitivity (Lee et al., 2002). Similarly, mutations in CHY1, which encodes a peroxisomal beta-hydroxyisobutyryl (HIBYL)-CoA hydrolase needed for valine catabolism and fatty acid beta-oxidation, also cause ROS accumulation and impair cold-responsive gene expression and freezing tolerance (Dong et al., 2009). Here, our data also showed It is majority of chloroplastic or mitochondrial related proteins were down-regulated, including Ketol-acid reductoisomerase, Glutamate decarboxylase, Malate dehydrogenase, Pentatricopeptide repeat-containing protein, Phosphoglycolate phosphatase, Monodehydroascorbate reductase, Aldehyde dehydrogenase family 2, Monodehydroascorbate reductase, Glycine dehydrogenase, and Probable mitochondrial-processing peptidase. Under cold stress, most aldehye dehydrogenase gene superfamily members showed decreased expression in grape and *Arabidopsis* (Zhang et al., 2012). Similar proteomic studies showed that some key enzymes involved in Krebs cycle (malate dehydrogenase) and many photosynthesis-related proteins (ATP synthase subunits) were down-regulated in wheat exposed to cold stress (Li et al., 2015). In addition, the accumulation of stress defense proteins including Cu/Zn superoxide dismutase, ascorbate peroxidases were significantly increased in bread wheat exposed to cold stress (Han, Kang & Guo, 2013). Many previous studies are consistently showed that GO terms related to photosynthesis and CO_2_ fixation are down-regulated as well (Sanz-Saez et al., 2010; Kazemi-Shahandashti & Maali-Amiri, 2018; Yu et al., 2018). More and more evidence showed that chloroplast and photosynthesis are affected when plants subjected to cold stress. The similar results of the reduction of photosynthesis, which we found in pitaya, were also reported in maize. The accumulation of chlorophyl in actively glowing rice leaves was significantly inhibited in cold stress (Glaszmann, Kaw & Khush, 1990). This reduction of photosynthesis might relate to the phenomenon that low temperature conditions cause a reduction in maximum quantum yields for CO_2_ uptake, as well as reduce the photochemical efficiency of photosystem II and then result to decrease the rate of light saturated photosynthesis (Kratsch & Wise, 2000; Renaut, Hoffmann & Hausman, 2005). A proteomics study of *Thellungiella halophila* based on two-dimensional electrophoresis demonstrated that half of differential abundant proteins stimulated under cold stress were identified to related to chloroplast physiology and function (Gao et al., 2009), which also suggesting that partial of cold stress tolerance regulation is going through chloroplast function or metabolism. A comparable analysis of rice seeding proteome also gave similar results (Hashimoto & Komatsu, 2007), further corroborating our pitaya proteomics data.

## Conclusion

The RNA-Seq technology is an efficient technology for characterizing transcriptomes of the non-model organisms. In this study, the Illumina paried-end sequencing project generated a substantial fraction of pitaya genes can be used as the reference and further investigated the proteome. Label-free proteomic analysis study of pitaya identified 116 DAPs associated with the cold stress in pitaya, of which, 18 proteins were up-regulated and 98 proteins were down-regulated. KEGG analysis and other results showed that these DAPs mainly related to chloroplasts and mitochondria metabolism. In summary, chloroplasts and mitochondrial metabolism-related proteins may play an important role in response to cold stress in pitaya.

## Supplemental Information

10.7717/peerj.8540/supp-1 Supplemental Information 1 List of genes identified by RNAseq.

10.7717/peerj.8540/supp-2 Supplemental Information 2 List of proteins identified by proteome study.

10.7717/peerj.8540/supp-3 Supplemental Information 3 Significant expressed proteins.

10.7717/peerj.8540/supp-4 Supplemental Information 4 Peptide sequences from proteome study.

10.7717/peerj.8540/supp-5 Supplemental Information 5 CDS sequences from RNAseq.

10.7717/peerj.8540/supp-6 Supplemental Information 6 Unigene sequences from RNAseq.

## Additional Information and Declarations

### Data Availability

The following information was supplied regarding data availability: All RNAseq and proteomic data files are available from the Integrated Proteome Resources: IPX0001296002.
